# Significant reduction of cognitive distortions after total sleep deprivation in patients with major depression: a preliminary study

**DOI:** 10.1192/j.eurpsy.2025.1358

**Published:** 2025-08-26

**Authors:** S. Poletti, G. D’Orsi, M. Carminati, R. Zanardi, C. Colombo, F. Benedetti

**Affiliations:** 1 IRCCS Ospedale San Raffaele, Milano, Italy

## Abstract

**Introduction:**

Cognitive distortion is a central feature of depression, encompassing dysfunctional personality styles and attitudes, and negative thinking. One of the cognitive schemas characteristics of depressed individuals is the tendency to overestimate causal responsibility for negative events, but not for positive ones. Total sleep deprivation (TSD) has been shown to cause rapid and sustained antidepressant effects in depressed patients and to revert the biased self description present in these patients.

**Objectives:**

The aim of the study was to investigate if TSD treatment would change cognitive distortion in a sample of patients diagnosed with major depressive disorder (MDD).

**Methods:**

**Seven** patients with MDD (all females), completed the Cognition Questionnaire (CQ) to assess cognitive distortions and were assessed before and after TSD treatment. TSD protocol involved three cycle of sleep deprivation, each one lasted 36 hours and was followed by a night of recovery sleep. Light therapy was administered for 30 minutes at 3 am during waking nights and in the morning after the recovery sleep.

**Results:**

A significant reduction of the depressive symptomatology was observed at both objective (Hamilton depression rating scale -HDRS F=9.85, p=0.008) and subjective (Beck depression Inventory – BDI F=54.73, p<0.001) measures. Investigating the 5 dimensions assessed through the CQ, we observed that the reduction of the dimension “attribution of causality” ( r=-7707, p=0.043) and the total CQ score (r=-.8865, p=0.008) after TSD were significantly associated with the reduction of depressive symptomatology as measured by the BDI.

**Conclusions:**

This is a preliminary study on the effect of TSD treatment on cognitive distortion in MDD. TSD treatment improved not only clinical symptoms but also cognitive distortion. Our preliminary findings suggest that TSD could be added to antidepressant treatment to rapidly improve the depressive symptomatology and cognitive distortion which may hamper the compliance with pharmacologic treatments and consequently the reduce the possibilities of a favouble outcome of pharmachologic interventions.

**Disclosure of Interest:**

None Declared

